# Multimodal machine learning for menopause status prediction using LLM-extracted ultrasound features

**DOI:** 10.3389/fendo.2026.1869205

**Published:** 2026-07-02

**Authors:** Weiwei Yin, Zhengyuan Shen, Chun Feng, Xia Zhang, Sihao Shen, Yiyue Jiang, Zhenbo Cheng, Lihui Wang, Ling Liu

**Affiliations:** 1 Department of Obstetrics and Gynecology, Hangzhou Red Cross Hospital, Hangzhou, Zhejiang, China; 2 College of Computer Science and Technology and College of Software, Zhejiang University of Technology, Hangzhou, Zhejiang, China; 3 The Second Affiliated Hospital Zhejiang University School of Medicine, Hangzhou, Zhejiang, China

**Keywords:** data mining, large language model, machine learning, menopause risk prediction, multimodal fusion

## Abstract

**Background:**

The assessment of menopausal status is crucial for individualized health management and risk stratification of chronic diseases in women. The traditional assessment of menopause mainly relies on serum reproductive hormone tests, such as FSH, AMH, etc., whose test results are stable and reliable and are the gold standard for clinical diagnosis. Meanwhile, ultrasound is a routine gynecological examination method. But the unstructured information in its report has not been systematically used for predictive modeling.

**Methods:**

This study included 713 Chinese women from Hangzhou Red Cross Hospital as the training set and another 284 from the Second Affiliated Hospital of Zhejiang University as the independent external validation set. Three morphological features of ovarian atrophy (OA), endometrial atrophy (EA) and uterine atrophy (UA) were automatically extracted from unstructured ultrasound report text by using large language model (LLM), and fused with anthropometric features to construct a multimodal prediction model. Eight machine learning models were trained and their predictive performances were compared.

**Results:**

The highest AUC of 0.984 was obtained by integrating anthropometric and hormone features in the validation set. The AUC is 0.935, which combines anthropometric features and ultrasound morphological features. The qwen-plus with the largest number of LLM parameters has the best feature extraction performance and is closest to the labeling results of human experts.

**Conclusion:**

LLM can effectively extract structured morphological features from ultrasound report text. The fusion of ultrasound morphological features and anthropometric features can provide supplementary evaluation methods for assessing menopausal status. This method is particularly suitable for clinical scenarios where hormone data is temporarily missing or unavailable.

## 1 Introduction

Menopause is a crucial physiological turning point in women’s lives, marking a permanent decline in ovarian function and a significant decrease in estrogen levels. Accurate prediction of menopausal status during perimenopause has significant clinical significance (1, 2). From the perspective of personal health management, accurate prediction can timely initiate hormone replacement therapy within the optimal treatment window, reducing the risk of osteoporosis and cardiovascular diseases associated with long-term estrogen deficiency (3). From a public health perspective, during the perimenopausal transition period, patient symptoms overlap with thyroid dysfunction, depression, and menstrual disorders, and diagnostic uncertainty may lead to inappropriate treatment decisions (4, 5). Currently, multiple guidelines suggest using hormone assessment as a diagnostic (6). However, especially for women with atypical manifestations or missing hormone data, integrating supplementary morphological information from routine ultrasound examinations can improve diagnostic confidence. Clinical menopausal assessment can be conducted using structured data such as hormones and anthropometry alone, or in combination with unstructured ultrasound report text. Therefore, developing a predictive model that is compatible with various data input forms and has stable and reliable predictive performance has important clinical value.

Currently, international clinical practice guidelines consistently endorse hormone testing as the diagnostic standard for menopause assessment. The STRAW (Stages of Reproductive Aging Workshop) (7) establishes FSH and E2 as key endocrine markers. Guidelines from the European Society of Endocrinology (ESE) (8), the UK’s National Institute for Health and Care Excellence (NICE) (9), and the Royal College of Obstetricians and Gynaecologists (RCOG) (10) reaffirm the central role of hormonal assessment in conjunction with symptomatic evaluation. AMH, due to its direct correlation with ovarian reserve, has been regarded as a sensitive indicator for predicting the time window of menopause in recent years (11, 12). FSH exhibits physiological variability during the perimenopausal transition, and clinical correlation is often beneficial (13).

Transvaginal ultrasound, as a routine gynecological examination method, can provide objective measurements of ovarian volume, antral follicle count, endometrial thickness, and uterine size. Previous studies have confirmed that the endometrial thickness of postmenopausal women is usually less than 5mm, while the proliferative endometrium of reproductive-aged women can reach 8–14mm (14, 15). The volume of the ovaries significantly decreases with age, and after menopause, the ovaries are often difficult to clearly visualize (16, 17). Although these morphological features have good discriminatory value, traditional methods rely on manual interpretation by ultrasound doctors. There has been no previous research on systematically transforming these unstructured texts into structured features that can be used for machine learning modeling.

LLM have demonstrated outstanding ability in extracting structured features from unstructured medical texts. GPT-4 has been successfully applied to extract structured information from histopathological reports without the need for specific task training (18). In the field of radiology, commercial LLMs such as GPT-4 and ChatGPT-3.5 have shown good performance in converting free text reports into structured formats (19). In addition, open-source LLMs like Vicuna have been proven to be able to automatically tag radiology reports for local deployment without exposing sensitive patient data (20). These studies collectively demonstrate that LLM based structured feature extraction is feasible in various medical fields.

Although LLM have demonstrated excellent performance in extracting medical text information, the existing research still has the following key shortcomings: Firstly, the morphological descriptions in ultrasound reports, such as ovarian atrophy, thinning of the endometrium, changes in uterine size, contain rich pathophysiological information related to menopause, but existing studies have failed to systematically convert these unstructured texts into structured features that can be used for machine learning modeling; Secondly, for the specific clinical task of predicting menopause status, the automatic extraction and verification of ultrasound features based on LLM have not been reported. The contributions of this study are as follows:

1. The application of LLM in automatic extraction of morphological features in ultrasound reports has verified that LLM can effectively mine unstructured medical text information.
2. This paper constructed a multimodal prediction framework that integrates anthropometric features, reproductive hormones, and ultrasound morphology features, which can maintain high prediction accuracy even when a certain type of feature is missing.
3. SHAP explanatory analysis elucidated the decision-making mechanism of the model and verified the role of ultrasound morphological features in assisting prediction.
4. This paper developed a web application for predicting the probability of menopause in perimenopausal women.

The remainder of this paper is organized as follows: Section 2 introduces Materials and Methods; Section 3 presents experimental results; Section 4 provides discussion; Section 5 concludes the study.

## 2 Materials and methods

### 2.1 Study population

This study employed a retrospective cohort design and included female subjects from two independent centers. Data were collected between January 2020 and April 2026. The training set came from Hangzhou Red Cross Hospital, consisting of 713 cases enrolled from January 2020 to April 2025. The external validation set came from the Second Affiliated Hospital of Zhejiang University School of Medicine, with a total of 284 cases collected from April 2025 to April 2026. A standardized preprocessing pipeline was applied uniformly across both centers to eliminate site-specific variability. All hormone measurements were collected during the early follicular phase (days 2–5) for premenopausal women, while samples from postmenopausal women were obtained at random time points. The study was approved by the Medical Ethics Committee of Hangzhou Red Cross Hospital (Approval No. 2026-053-001) and the Ethics Committee of the Second Affiliated Hospital of Zhejiang University School of Medicine (Approval No. 2026-0688). All procedures were conducted in accordance with the Declaration of Helsinki. Due to the retrospective nature of the study, the requirement for informed consent was waived by the ethics committees.

In order to focus on the menopausal transition period with the highest diagnostic ambiguity, the training was only conducted on subjects aged 45-55. This age restriction resulted in 305 cases in the training set and 110 cases in the external validation set. The baseline clinical characteristics of the model development queue are detailed in **Table 1**.Where missing features are filled with means.

**Table 1:** Baseline features of study population.

Feature	Missing	PreM	PostM	Missing	PreM	PostM
	Rate	(n=535)	(n=178)	Rate	(n=205)	(n=79)
Age (years)	0.00%	40.14 ± 7.95	55.06 ± 7.00	0.00%	41.00 ± 7.34	57.70 ± 6.78
Height (m)	0.28%	1.60 ± 0.05	1.59 ± 0.05	0.00%	1.60 ± 0.05	1.58 ± 0.05
Weight (kg)	0.28%	58.72 ± 9.59	60.56 ± 8.18	0.00%	59.37 ± 9.01	59.84 ± 8.48
FSH (mIU/mL)	0.70%	7.46 ± 6.16	51.47 ± 23.99	0.00%	8.73 ± 8.13	61.20 ± 28.36
LH (mIU/mL)	0.14%	8.41 ± 9.37	26.15 ± 14.94	0.00%	8.21 ± 9.64	29.44 ± 15.08
P (ng/mL)	0.14%	3.14 ± 6.15	0.47 ± 0.98	0.00%	2.31 ± 4.06	0.30 ± 0.27
E2 (pg/mL)	0.14%	126.77 ± 138.45	24.93 ± 46.48	0.00%	110.02 ± 105.11	12.79 ± 14.90
Prolactin (ng/mL)	0.56%	13.51 ± 8.36	8.72 ± 5.05	0.00%	13.80 ± 9.21	9.42 ± 7.20
Testosterone (ng/mL)	0.56%	0.37 ± 0.21	0.29 ± 0.19	0.00%	0.34 ± 0.24	0.24 ± 0.19
AMH (ng/mL)	54.14%	2.01 ± 2.22	0.02 ± 0.07	63.38%	1.56 ± 1.42	0.01 ± 0.01
BMI (kg/m^2^)	0.28%	22.78 ± 3.41	23.97 ± 3.02	0.00%	23.21 ± 3.30	24.04 ± 2.95
OA	3.65%	–	–	7.39%	–	–
EA	15.71%	–	–	15.85%	–	–
UA	7.57%	–	–	8.45%	–	–

All ultrasound examinations were performed using standardized gynecological protocols at both centers. At Hangzhou Red Cross Hospital, examinations were conducted using Philips EPIQ 7 ultrasound systems with 5–9 MHz transvaginal probes. At the Second Affiliated Hospital of Zhejiang University, examinations were performed using the same equipment. Both centers utilized similar equipment specifications and imaging protocols, with routine quality assurance procedures in place. All ultrasound reports were generated by attending radiologists or gynecologists with subspecialty training in pelvic imaging.

**Figure 1:**
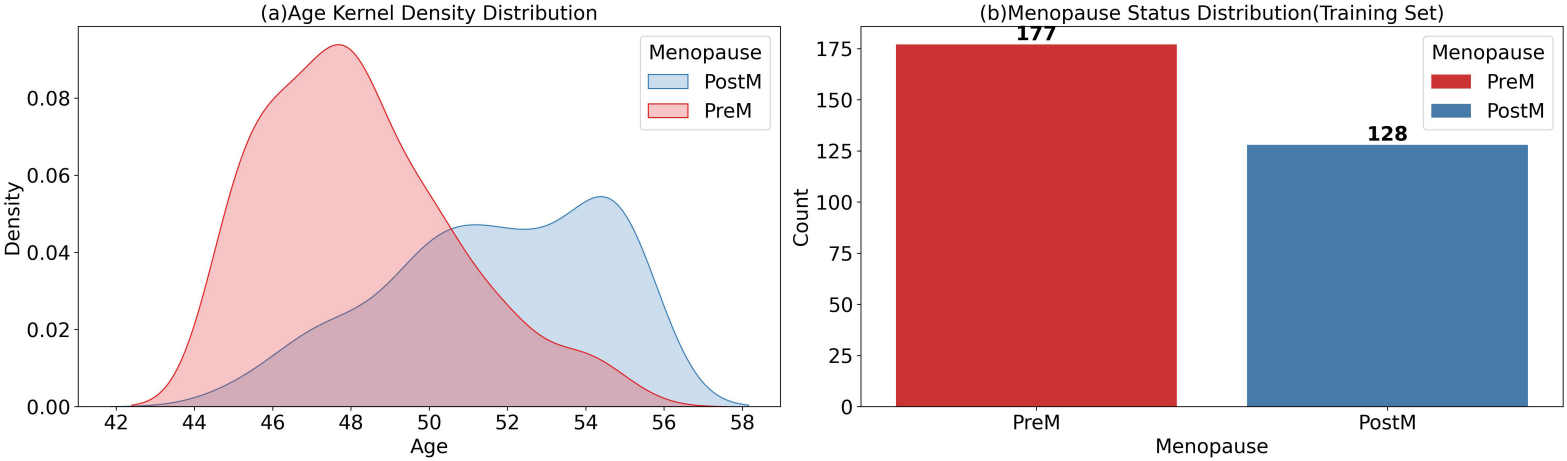
Age distribution and category composition of study population. **(a)** Age Kernel Density Distribution; **(b)** Menopause Status Distribution (Training Set).

In terms of anthropometric features, the postmenopausal women in both sets were slightly shorter than premenopausal women, while their weights and BMI significantly increased, reflecting the changes in body composition of postmenopausal women, including redistribution of fat and reduction in muscle mass. In the training set, there was a significant difference in age distribution between the two groups of subjects: the premenopausal group had an average age of 41 years, while the postmenopausal group was 55 years old, which was in line with the age distribution pattern of natural menopause in women. As shown in **Figure 1**, the premenopausal group in the training set showed a unimodal age distribution with a peak in the 35–45 year range; the postmenopausal group exhibited a wider distribution range, mainly concentrated between 50–65 years.

### 2.2 Diagnostic, inclusion, and exclusion criteria

Diagnostic criteria for this study: Criteria for determining menopause: According to the natural menopause standard, absence of menstrual periods for 12 consecutive months, excluding factors such as pregnancy, lactation, ovarian surgery, radiotherapy, and chemotherapy due to medical intervention, and a comprehensive determination based on clinical diagnosis and hormone levels.

Inclusion criteria: (1) Female patients aged 20–80 years; (2) Height and weight records available at the hospital for BMI calculation; (3) All six reproductive hormone tests were completed simultaneously. AMH, as a supplementary indicator for assessing ovarian reserve function, was selectively tested based on clinical needs; (4) Menopause status could be clearly determined from clinical data; (5) Clinical data was traceable and met requirements for retrospective study use.

Exclusion criteria: (1) History of iatrogenic interventions affecting menopause status determination, such as oophorectomy, hysterectomy, or pelvic radiotherapy/chemotherapy; (2) Use of sex hormones, ovulation induction drugs, or hormonal contraceptives within the past 3 months that could interfere with endocrine indicators; (3) Suffering from severe endocrine or metabolic diseases such as polycystic ovary syndrome, premature ovarian failure, or thyroid disease; (4) Pregnant or lactating women; (5) Missing clinical data, inability to determine menopause status, or uncorrectable data anomalies; (6) Concurrent severe liver or kidney dysfunction or major physical diseases such as malignant tumors that could affect hormone levels.

### 2.3 LLM feature extraction

**Figure 2:**
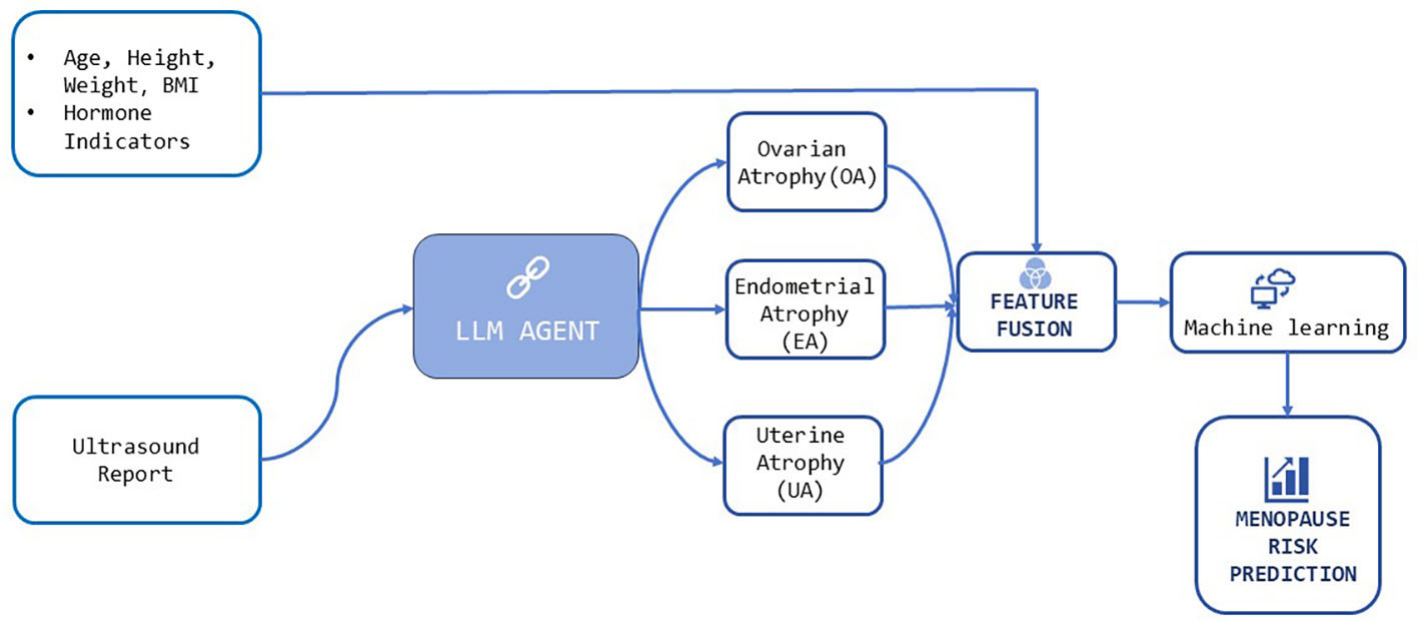
LLM feature extraction workflow and machine learning training process.

This study utilized LLM to automatically extract menopause-related morphological features from unstructured ultrasound report text, transforming free-text ultrasound descriptions into structured features suitable for machine learning modeling. The overall workflow is shown in **Figure 2**.

This study constructs an LLM feature extraction pipeline based on the LangChain framework and calls the Qwen3.5-plus LLM to perform structured parsing of ultrasound report text. Adopting a multi-threaded concurrent call strategy to achieve efficient and automated processing of large-scale ultrasound reports.

Based on menopause-related pathophysiological knowledge, we defined three categories of key morphological features: OA refers to ovarian volume reduction; EA refers to endometrial atrophy; UA refers to uterine volume reduction. As shown in Equation 1, the LLM maps ultrasound report text to three categorical variables:

(1) $$f_{\text{LLM}}:T\to {\left\{0,1,2\right\}}^{3}$$

where T represents the ultrasound report text space, and output values indicate: 0 for normal, 1 for abnormal (atrophy/thinning/smaller size/enhanced echogenicity/unclear visualization/not visualized), and 2 for no data extracted. The extracted morphological features are represented as a vector $y_{\text{ultrasound}}={\left[\text{OA},\text{EA},\text{UA}\right]}^{⊤}$.

This paper designs a structured prompt template to assign the role of ultrasound expert to LLM, providing clear classification definitions and decision boundaries for each feature, and enforcing strict JSON output patterns for integer values in {0,1,2}. Three manual few shot examples are directly embedded into the prompt to standardize the inference mode. The complete prompt template and corresponding implementation code can be accessed through our GitHub repository, as stated in the data availability statement.

Multimodal feature fusion adopts an early fusion strategy, As shown in Equation 2, multimodal feature concatenating the clinical demographic feature vector $x_{\text{demo}}\in {\mathbb{R}}^{4}$, reproductive hormone feature vector $x_{\text{hormone}}\in {\mathbb{R}}^{7}$, and LLM-extracted ultrasound morphological feature vector $y_{\text{ultrasound}}\in {\mathbb{R}}^{3}$:

(2) $$x_{\text{fused}}=x_{\text{demo}}⊕x_{\text{hormone}}⊕y_{\text{ultrasound}}$$

where ⊕ represents the vector concatenation operation, and $x_{\text{fused}}\in {\mathbb{R}}^{14}$ is the fused multimodal feature vector, serving as input for subsequent machine learning models.

### 2.4 Model development and validation

In this study, eight machine learning models were used to construct a menopausal status prediction model, including XGBoost, Random Forest (RF), Support Vector Machine (SVM), K-Nearest Neighbors (KNN), LightGBM, Artificial Neural Network (ANN), CatBoost, and Naive Bayes (NB). All models were trained on the training set and independently evaluated on the external validation set. Model performance was assessed using AUC, accuracy, precision, recall, and F1 score, with ROC curves plotted. All hyperparameter are defaulted. Model interpretability analysis employed the SHAP (SHapley Additive exPlanations) method to quantify each feature’s contribution to model predictions (21).

## 3 Results

### 3.1 Reproductive hormone features

**Figure 3:**
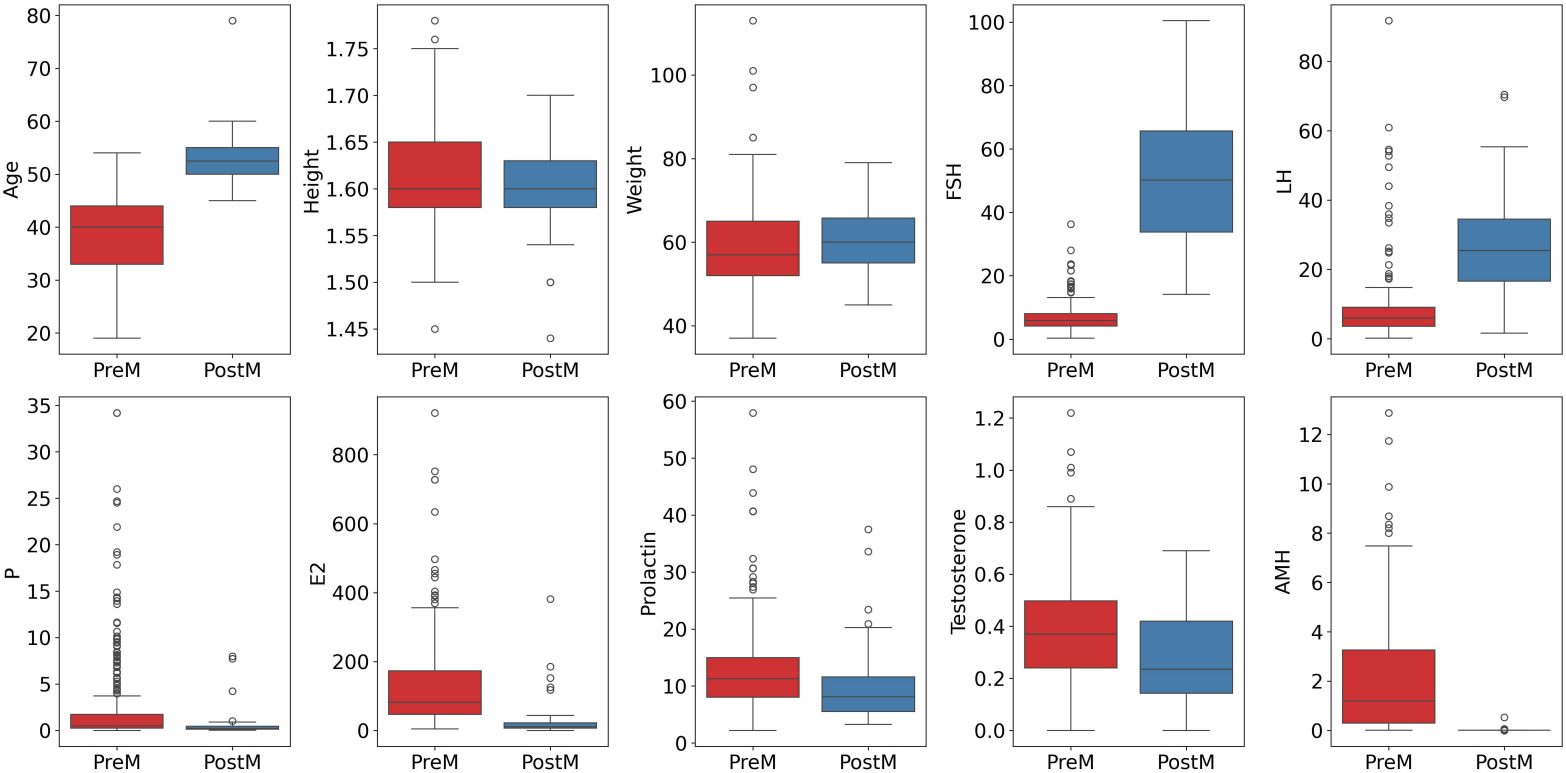
Box plots of reproductive hormone level comparisons between groups. Postmenopausal women showed significantly elevated FSH and LH, and significantly reduced E2 and AMH, reflecting HPO axis function decline.

Reproductive hormone levels demonstrated marked changes between premenopausal and postmenopausal women (**Figure 3**), consistent with HPO axis functional decline. FSH and LH increased substantially (51.47 vs 7.46 mIU/mL and 26.15 vs 8.41 mIU/mL, respectively), while E2 decreased by 88% (12.79 vs 110.02 pg/mL). AMH showed the largest relative reduction (≥99%, 0.02 vs 2.01 ng/mL), though with a high missing rate (54.14%) due to its selective use primarily in fertility assessments. These patterns align with established physiological changes of menopause.

### 3.2 Ultrasound morphological features

**Figure 4:**
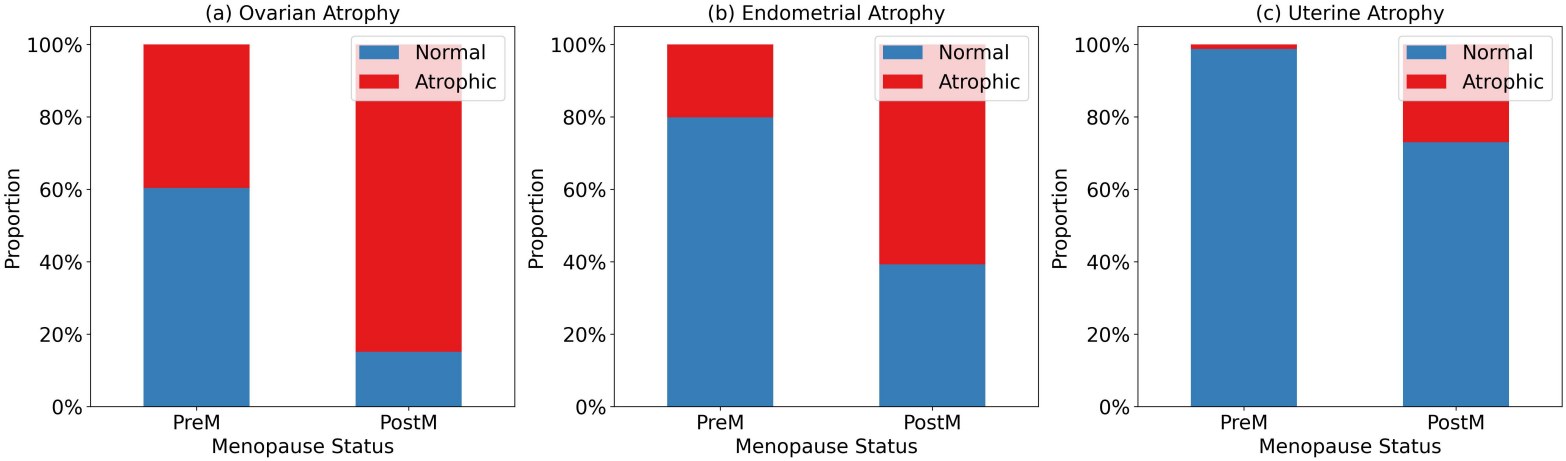
Extract ultrasound morphological features through LLM. **(a)** Ovarian Atrophy; **(b)** Endometrial Atrophy; **(c)** Uterine Atrophy.

This study utilized LLM to perform natural language processing on ultrasound reports and extracted three types of morphological features. The distribution of these features among groups is shown in **Figure 4**.

OA is a characteristic morphological change during menopause. As shown in **Figure 4a**, the incidence of ovarian atrophy significantly increases in postmenopausal women. The size of the ovaries decreases and follicles are depleted after menopause, making it difficult to clearly visualize them under ultrasound. The ovarian atrophy features extracted by LLM in this study can effectively capture this menopause-related morphological change.

EA is a sensitive feature for evaluating the biological effects of estrogen. As shown in **Figure 4b**, the incidence of endometrial atrophy in postmenopausal women is significantly higher than that in premenopausal women. This finding is consistent with the reduction in endometrial proliferative activity caused by estrogen deficiency.

UA reflects the nutritional support role of estrogen on uterine smooth muscle cells. As shown in **Figure 4c**, This structural change is associated with decreased expression of estrogen receptors and increased apoptosis of myometrial cells.

In summary, all three morphological features extracted from ultrasound reports by LLM showed significant intergroup differences and were highly correlated with menopause status. These features complement reproductive hormone features, together forming the multimodal data foundation for menopause assessment.

### 3.3 Feature correlation analysis

**Figure 5:**
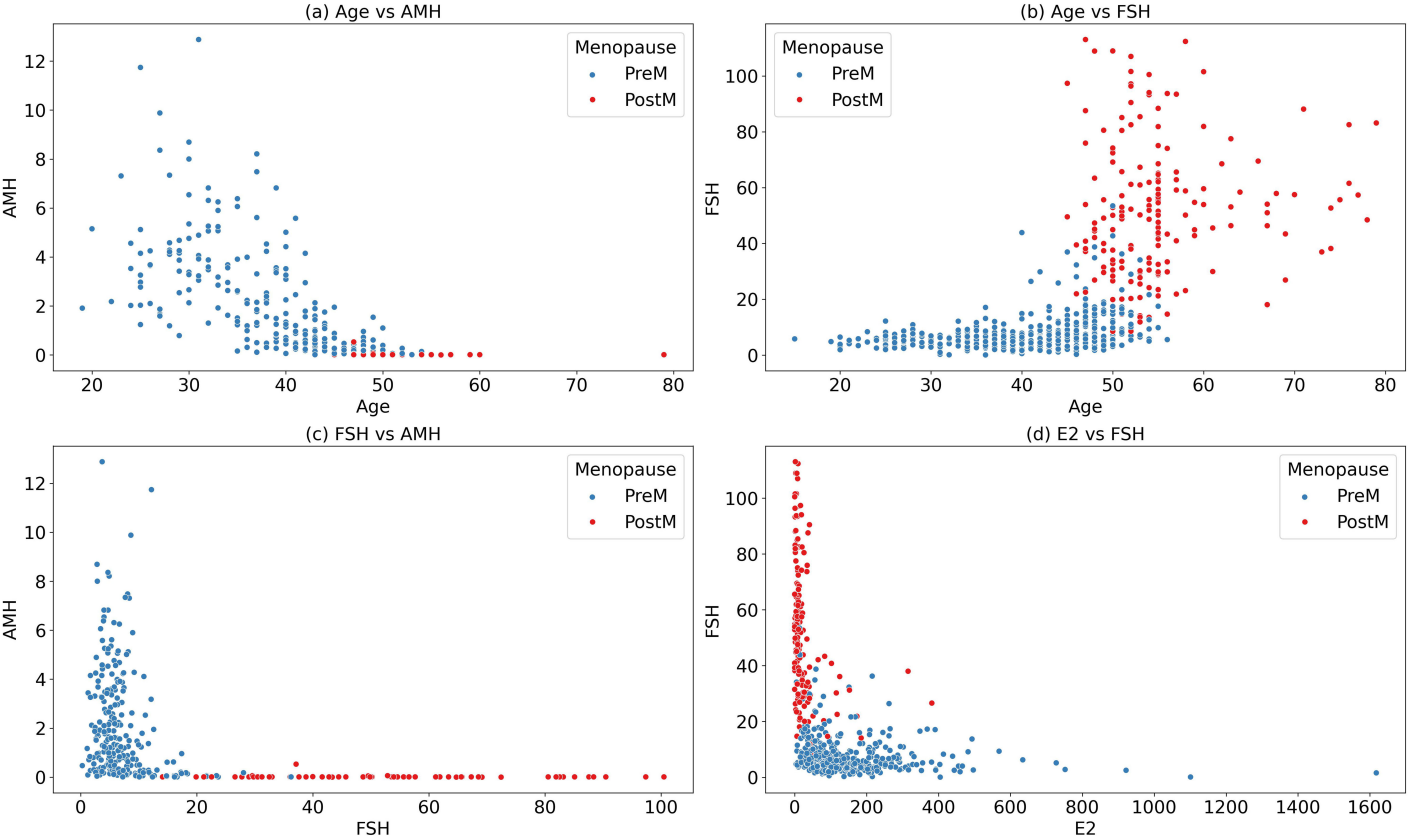
Joint distribution scatter plots of key reproductive hormone features. Each point represents a subject. **(a)** correlations between age and AMH; **(b)** correlations between age and FSH; **(c)** correlations between FSH and AMH; **(d)** correlations between E2 and FSH.

Age is significantly negatively correlated with AMH and positively correlated with FSH, which is in line with the physiological rule that ovarian reserve function declines with age. **Figure 5a** shows that the AMH level is relatively stable before the age of 35, then accelerates to decline, and drops below the detection limit around the age of 50. This non-linear decline trajectory is consistent with the index model of follicle exhaustion. **Figure 5b** shows that FSH significantly increases after the age of 45, reflecting the compensatory secretion increase of pituitary gonadotropins during the menopausal transition period.

**Figure 5c** shows a significant negative correlation between the two features, further supporting the bidirectional assessment mechanism of ovarian reserve function. AMH can directly reflect the level of ovarian reserve, while FSH indicates the compensatory secretion changes of the pituitary gland. Together, they can form an effective complement in the assessment of menopausal-related conditions.

FSH showed a strong positive correlation with LH, and both were negatively correlated with E2, reflecting the endocrine negative feedback regulation between the pituitary gland and ovary. **Figure 5d** clearly demonstrates this negative feedback relationship: when the E2 level is below 50 pg/mL, the FSH level significantly increases, forming a typical negative correlation curve. This relationship is consistent with the physiological regulatory mechanism of the HPO axis - the E2 secreted by the ovary inhibits the secretion of FSH by the pituitary gland through negative feedback. When ovarian function declines and the E2 level drops, the negative feedback is released, and FSH compensatorily increases.

### 3.4 Model performance

**Figure 6:**
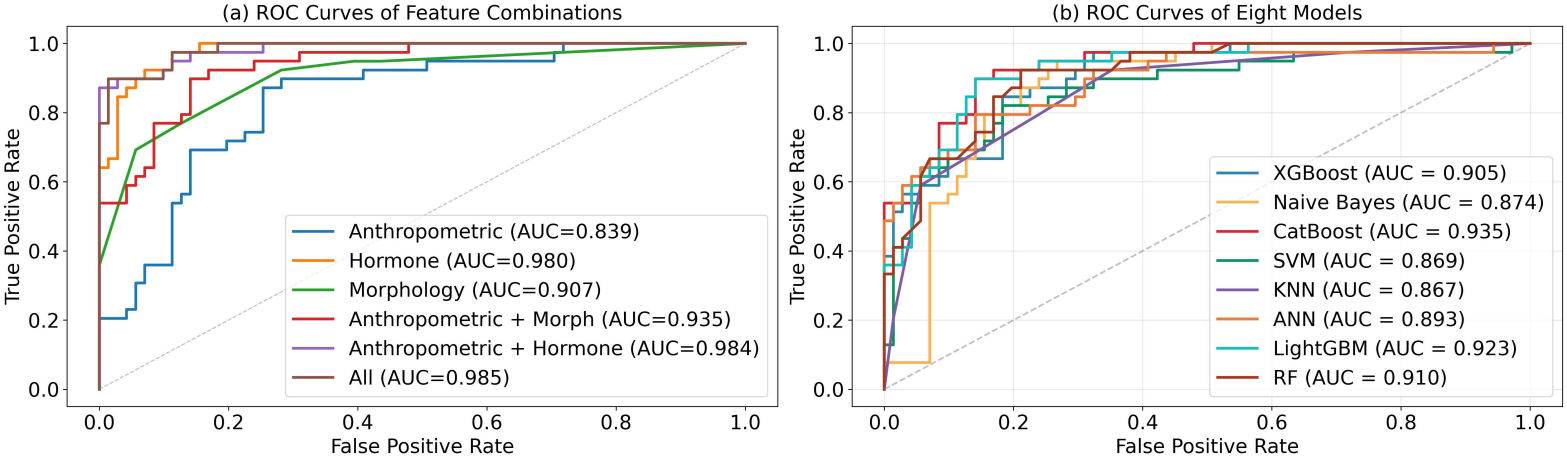
Multimodal feature model performance comparison. **(a)** Comparison of prediction performance of different feature combinations on Catboost model;**(b)** Comparison of eight models.

In order to evaluate the contribution of ultrasound morphological features extracted by LLM to menopausal state prediction, this study first compared the model performance when different feature combinations were trained on a training set and evaluated on an external validation set. As shown in **Figure 6a**, ROC curve analysis indicates that the AUC obtained using only anthropometric features is 0.839; The AUC generated using only reproductive hormone features is 0.980; The AUC obtained using only ultrasound morphological features is 0.907. The AUC obtained by combining anthropometric and ultrasound morphological features is 0.935. The highest AUC obtained by merging anthropometric and hormonal features was 0.984.The AUC trained using all features is 0.985. The experimental results indicate that the performance of hormone features is the best, followed by ultrasound morphological features. The anthropometric features have a certain amplification effect on the prediction results. Ultrasound features are a supplement to hormone feature prediction and can play a role when patients lack hormone features.

In the external validation set, we further evaluated the generalization performance of eight machine learning models. As shown in **Figure 6b** and **Table 2**, all models demonstrated good predictive performance in the external validation set. Among them, the CatBoost model performed the best, with an AUC of 0.9354 and an F1 score of 0.8333. Next are LightGBM, RF, and XGBoost. By comprehensively comparing the performance of 8 models, the results of this study indicate that the ensemble learning method based on tree models is generally superior to traditional machine learning methods in predicting menopausal states, and has good cross center generalization ability.

**Table 2:** Model performance.

Model	AUC	Accuracy	Precision	Recall	F1 Score
XGBoost	0.9047	0.8	0.6977	0.7692	0.7317
LightGBM	0.9231	0.8545	0.7805	0.8205	0.8
RF	0.9101	0.8364	0.7333	0.8462	0.7857
CatBoost	0.9354	0.8727	0.7778	0.8974	0.8333
ANN	0.8931	0.8273	0.75	0.7692	0.7595
SVM	0.8693	0.8182	0.8276	0.6154	0.7059
KNN	0.8673	0.7727	0.6458	0.7949	0.7126
Naive Bayes	0.8736	0.8	0.6809	0.8205	0.7442

To validate the reliability of LLM-extracted morphological features, 100 ultrasound reports were randomly selected from the training set and independently interpreted by two attending gynecologists. The LLM extraction results were compared against the expert consensus to ensure clinical validity. This paper also compared the performance of LLMs with different parameter sizes in ultrasound morphological feature extraction tasks, and the results showed a positive correlation between model size and extraction performance. As shown in **Table 3**, the qwen-plus with the largest parameter size performs the best in OA, UA. The qwen-flash has a slight advantage in extracting endometrial EA with an F1 score of 0.8824, while the qwen-turbo with the smallest parameter size has relatively lower F1 score in all aspects. The above results show that models with larger parameter scales have more stable advantages in semantic understanding of complex medical texts.

**Table 3:** Comparison of LLMs with different parameter sizes.

Model	OA F1 score	EA F1 score	UA F1 score	Macro F1 score
qwen-turbo	0.7306	0.8313	0.6799	0.7473
qwen-flash	0.7787	0.8824	0.8616	0.8409
qwen-plus	0.8657	0.8478	0.9230	0.8788

## 4 Discussion

### 4.1 SHAP interpretability analysis

To clarify the prediction mechanism of the model and identify the key predictive factors, this study employed the SHAP method to conduct global and local interpretability analyses of the optimal model. SHAP is based on the Shapley value in cooperative game theory, assigning a unique marginal contribution to each feature, and has a solid theoretical foundation and wide clinical applicability (21).

**Figure 7:**
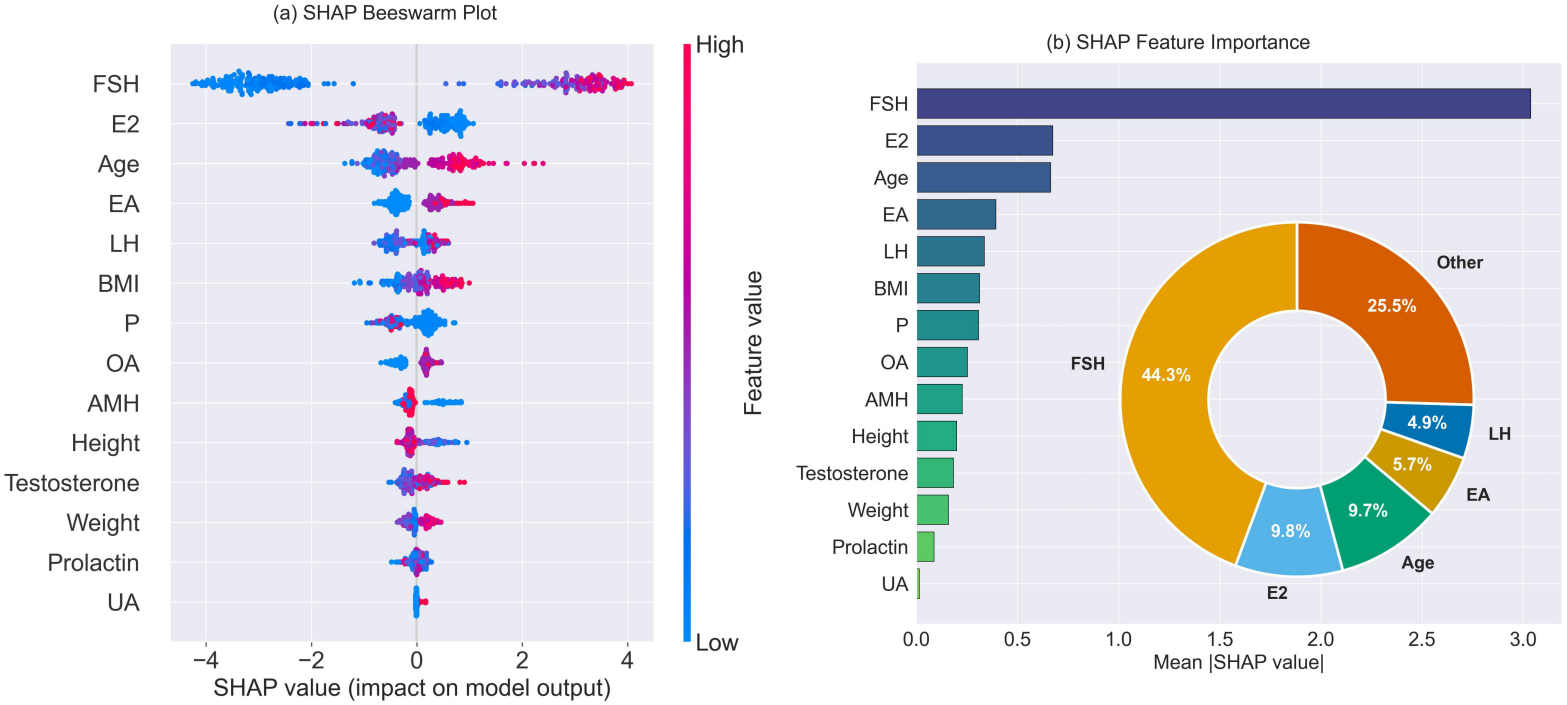
Anthropometric features, morphological features, and hormone features combination training. **(a)** Beeswarm plot showing contribution of each feature value to prediction, red indicates high feature values, blue indicates low feature values. **(b)** Feature importance ranking shows FSH as the most important predictive factor.

The beeswarm plot in **Figure 7a** shows the distribution relationship between the feature values of each sample and their SHAP values under the complete feature combination. It can be seen that high FSH, high LH, high age, low AMH, and low E2 levels have positive contributions to the prediction of menopause, while low FSH, high AMH, and low age have negative contributions. The direction of the effect of these features is completely consistent with the physiological mechanism of negative feedback regulation of the HPO axis (22, 23). The feature importance ranking in **Figure 7b** shows that the SHAP importance of FSH is much higher than that of all other features, confirming the core role of FSH in the discrimination of menopausal status, which is consistent with the traditional understanding in clinical practice that it is regarded as the gold standard for diagnosing menopause (24). estradiol (E2) ranks second in importance, while the importance of age, LH, BMI, and AMH follows closely, suggesting that although this information is not the primary driving factor, it still contributes to the incremental discriminative information.

**Figure 8:**
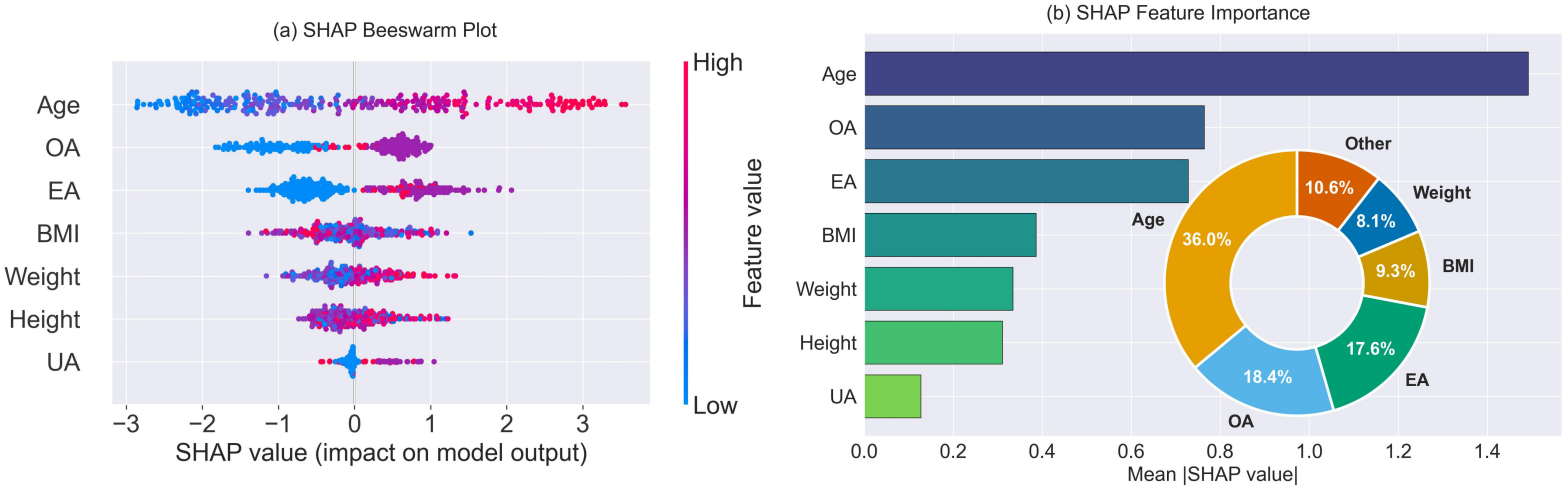
Training with only anthropometric features and morphological features. **(a)** Beeswarm plot showing contribution of each feature value to prediction, red indicates high feature values, blue indicates low feature values. **(b)** Feature importance ranking shows Age as the most important predictive factor.

The beeswarm plot in **Figure 8a** shows the relationship between each feature and the SHAP value when only anthropometric and morphological features are considered. Compared with the complete feature combination, when hormone data is missing, age replaces FSH as the most important predictor, which is in line with the clinical understanding that menopause is an age-related physiological process (12). In terms of ultrasound morphological features, OA, EA, UA all show positive SHAP contributions, indicating that the presence of these structural changes significantly increases the probability of predicting menopause; conversely, normal morphology has a negative contribution to the prediction of menopause. Despite using only anthropometric and morphological features, the model still achieved an AUC of 0.935, verifying the feasibility of combining age and ultrasound morphological features for menopause screening in the absence of hormone data.

### 4.2 Application of the web calculator

**Figure 9:**
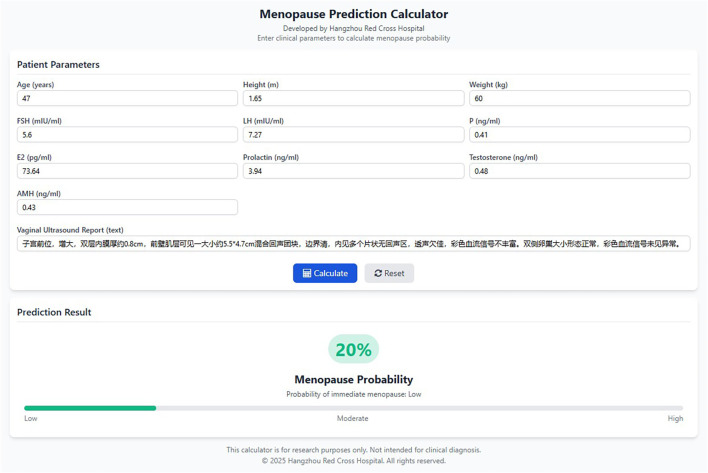
Multimodal menopause risk prediction web application interface. The system automatically outputs menopause probability prediction results.

The final prediction model developed in this study has been integrated into a network application (**Figure 9**), providing convenient menopausal state risk assessment services for perimenopausal women, as shown in the figure. Users only need to input anthropometric features such as age, height, and weight, actual values of reproductive hormone characteristics including FSH, LH, P, E2, prolactin, testosterone, and AMH, as well as ultrasound report text. Even if 1–2 categories are missing from the measurement features, ultrasound morphology features, and hormone features, the application can still output the probability of menopausal status. This helps achieve early screening of menopausal status and personal health management.

### 4.3 Key findings

This study utilized the qwen plus model to automatically extract OA, EA, and UA from unstructured gynecological ultrasound report text. The experimental results show that the LLM extraction results are highly consistent with manual expert annotation. The qwen plus model with the largest number of parameters achieved a Macro F1 score of 0.8788, which is better than models with smaller parameter scales. Confirmed the positive correlation between model size and medical text semantic understanding ability. This discovery provides empirical support for the application of LLM in clinical medical text information mining.

When using the ultrasound morphological features extracted by LLM alone for prediction, an AUC of 0.907 can be obtained on the external validation set, which is significantly higher than the 0.839 obtained by using human measurement features alone. When ultrasound morphological features are combined with anthropometric features, the AUC further increases to 0.935 and the F1 score reaches 0.8333. SHAP analysis shows that age, OA, EA, and UA all contribute positively to menopausal prediction in the absence of hormone data. This discovery confirms that in clinical settings where hormone data is temporarily missing or unavailable, morphological assessment based on routine ultrasound examination can serve as a reliable alternative evaluation method.

## 5 Conclusion

This article applies LLM to automatically extract morphological features from ultrasound reports, confirming that LLM can mine effective information in unstructured medical texts. Secondly, this study developed a multimodal menopausal prediction system that integrates anthropometric measurements, reproductive hormone indicators, and ultrasound morphological features. In addition, based on SHAP interpretability analysis, the internal predictive decision-making mechanism of the model was elucidated. Research has shown that ultrasound morphological features can serve as a supplement to hormone features for predicting menopause.

## Data Availability

The dataset can be obtained by contacting the corresponding author for reasonable reasons. And the experimental code and data analysis can be obtained from here: https://github.com/connorshen/USG-LLM.git.
